# Determinants of formation of aflatoxin-albumin adducts: a seven-township study in Taiwan

**DOI:** 10.1038/sj.bjc.6600584

**Published:** 2002-10-21

**Authors:** C-A Sun, D-M Wu, L-Y Wang, C-J Chen, S-L You, R M Santella

**Affiliations:** School of Public Health, National Defense Medical Center, School of Public Health, No. 161, Section 6, Min-Chuan East Road, Taipei 114, Taiwan, Republic of China; Institute of Aboriginal Health, Tzu Chi University, Hualien County 970, Taiwan, Republic of China; Graduate Institute of Epidemiology, College of Public Health, National Taiwan University, Taipei 100, Taiwan, Republic of China; Division of Environmental Health Sciences, Mailman School of Public Health, Columbia University, New York, NY 10032, USA

**Keywords:** aflatoxin-albumin adducts, glutathione S-transferase M1-1, glutathione S-transferase T1-1, hepatitis B surface antigen

## Abstract

Dietary exposure to aflatoxins is one of the major risk factors for hepatocellular carcinoma. Individual susceptibility to aflatoxin-induced hepatocarcinogenesis may be modulated by both genetic and environmental factors affecting metabolism. A cross-sectional study was performed to evaluate determinants of the formation of aflatoxin covalently bound to albumin (AFB_1_-albumin adducts). A total of 474 subjects who were free of liver cancer and cirrhosis and were initially selected as controls for previous case–control studies of aflatoxin-induced hepatocarcinogenesis in Taiwan, were employed in this study. Aflatoxin-albumin adducts were determined by competitive enzyme-linked immunosorbent assay, hepatitis B surface antigen and antibodies to hepatitis C virus by enzyme immunoassay, as well as genotypes of glutathione S-transferase M1-1 and T1-1 by polymerase chain reaction. The detection rate of AFB_1_-albumin adducts was significantly higher in males (42.5%) than in females (21.6%) (multivariate-adjusted odds ratio=2.6, 95% confidence interval=1.4–5.0). The formation of detectable albumin adducts was moderately higher in hepatitis B surface antigen carriers (42.8%) than in non-carriers (36.6%) (multivariate-adjusted odds ratio=1.4, 95% confidence interval=1.0–2.1). In addition, the detection rate of AFB_1_-albumin adducts tended to increase with the increasing number of null genotypes of *glutathione S-transferase M1-1* and *glutathione S-transferase T1-1*. In conclusion, this cross-sectional study has assessed the relative contributions of environmental exposure and host susceptibility factors in the formation of AFB_1_-albumin adducts in a well characterised Chinese adult population. This study further emphasises the necessity to reduce aflatoxin exposure in people living in an area endemic for chronic hepatitis B virus infection.

*British Journal of Cancer* (2002) **87**, 966–970. doi:10.1038/sj.bjc.6600584 www.bjcancer.com

© 2002 Cancer Research UK

## 

Pervious studies have indicated that hepatocellular carcinoma (HCC) is of multifactorial origin with both viral and chemical carcinogens involved in the multistage process. Although chronic infection with hepatitis B virus (HBV) is now regarded as the major cause of HCC in high-incidence areas (Yeh *et al*, 1989; Chen *et al*, 1991; Ryder *et al*, 1992), ingestion of aflatoxin B_1_ (AFB_1_) has also been implicated as another major contributor to risk (Yeh *et al*, 1989; Ross *et al*, 1992; Wang *et al*, 1996a,b). Many studies have confirmed that parent compound AFB_1_ is converted to its carcinogenic forms through metabolism by members of the endogenous cytochrome P-450 enzyme superfamily to reactive 8,9-epoxide metabolites, which can covalently interact with cellular DNA and proteins (International Agency for Research on Cancer, 1992; Gallagher *et al*, 1994; Guengerich *et al*, 1998). Among the major epoxide-derived macromolecular adducts identified, the AFB_1_-albumin adduct correlates well with other aflatoxin measurements and provides a cumulative measure of exposure over several months in humans (Wild *et al*, 1986; Gan *et al*, 1988).

Accumulating evidence indicates that susceptibility to aflatoxin-related HCC may be modulated by inter-individual differences in metabolism (Mcglynn *et al*, 1995; Chen *et al*, 1996b; Sun *et al*, 2001). Both epidemiological and experimental studies have demonstrated a synergistic interaction between chronic HBV infection and aflatoxin exposure in the development of HCC (Ross *et al*, 1992; Wang *et al*, 1996a; Sylla *et al*, 1999). Taken together, the degree that AFB_1_ contributes to risk of HCC may be influenced by both genetic and environmental factors. Thus, we performed a cross-sectional study of 474 control subjects enrolled in previous nested case–control study of susceptibility to aflatoxin-related HCC (Wang *et al*, 1996b; Sun *et al*, 2001) to evaluate the correlations of multiple HCC enviromental and genetic susceptibility risk factors with the formation of AFB_1_-albumin adducts in the peripheral blood.

## MATERIALS AND METHODS

### Study subjects

From July 1990 through June 1992, a community-based two-stage liver cancer screening programme was carried out in seven townships in Taiwan. The cohort characteristics and methods of screening and follow-up have been described previously (Wang *et al*, 1996b; Sun *et al*, 2001). Briefly, in the two-stage screening programme, study subjects aged 30 to 64 years were first screened by serological markers, including hepatitis B surface antigen (HBsAg), antibodies to hepatitis C virus (anti-HCV), alanine transaminase (ALT), aspartate transaminase (AST), and α-fetoprotein (AFP). HBsAg, anti-HCV, and AFP were tested by enzyme immunoassay using commercial kits (Abbott Laboratories, North Chicago, IL, USA), while both ALT and AST levels were determined by serum chemistry autoanalyzer (Hitachi Model 736; Hitachi Co., Tokyo, Japan) using commercial reagents (Biomerieux, Mercy I'Etoile, France). Any subject who had a positive status of HBsAg or anti-HCV, an elevated level of ALT (45 IU/L), AST (40 IU L^−1^), or AFP (20 ng ml^−1^), or a family history of HCC or liver cirrhosis among first degree relatives was referred for the second-stage screening by upper abdominal ultrasonography. The abdominal ultrasonography was performed by board-certified gastroenterologists who were well experienced in ultrasonographic examinations using Toshiba SAL-38B and SSA-240A ultrasonographic apparatus with 3.75 MHZ real-time linear and sector probes (Toshiba, Japan).

All participants were personally interviewed based on a structured questionnaire at recruitment. Blood samples were collected from each study subject. Aliquots of serum, buffy coat, plasma, and red blood cells were separated and stored at −70°C. Specimens were transported in dry ice to the central laboratory at the National Taiwan University and were kept in deep freezers until examination. All study subjects gave informed consent for both the interview and blood collection. In addition, anonymity of study subjects was maintained by the numerical coding of questionnaires and blood samples. This community-based cancer screening project was supported and approved by Department of Health, Executive Yuan.

The present study is concerned with 474 subjects who were initially selected as controls for previous nested case–control studies of aflatoxin-related hepatocarcinogenesis (Wang *et al*, 1996b; Sun *et al*, 2001). These individuals were not affected with HCC or cirrhosis through follow-up period. In fact, data used in the present study were obtained by mining of the original data set from these previous nested case-control studies.

### AFB_1_-albumin adducts in serum

An enzyme-linked immunosorbent assay was used to determine the level of AFB_1_-albumin adducts in serum as previously described (Wang *et al*, 1996a; Sun *et al*, 2001). This assay had 50% inhibition of antiserum binding at 10–20 fmol AFB_1_ adduct per well. The limit of sensitivity (20% inhibition) when assaying the equivalent of 200 μg albumin per well was 0.01 fmol μg^-1^. Samples were assayed by duplicate analysis in duplicate wells. Samples with <20% inhibition were considered non-detectable. Two control samples were analysed with each batch of sera, a pooled sample of plasma from non-smoking US subjects and a positive control of serum from a rat treated with 1.5 mg AFB_1_.

### *GSTM1-1* and *GSTT1-1* genotypes

*GSTM1-1* genotyping for gene deletion was performed by PCR amplification with primers for exons 6 and 7, which produced a 210 bp band, according to the method of Bell *et al* (1993). *GSTT1-1* genotype was determined using the technique of Pemble *et al* (1994), with the modification that β-globin primers were added to the PCR.

### Statistical methods

Because it was not considered appropriate to assign a value to the undetectable serum level of AFB1-albumin adducts, the adducts level was analysed as a binary rather than continuous variable. Odds ratios (OR) and their 95% confidence intervals (CI), which were derived from logistic regression models, were used to indicate the magnitude of the associations between formation of AFB_1_-albumin adducts and various variables. In addition, months of year for blood sample collection were grouped into four seasons in order to evaluate seasonal variations in the detectable levels of AFB_1_-albumin adducts. All analyses were performed with SAS software (SAS Institute, Cary, NC, USA) and all *P* values for tests of statistical significance were based on two-tailed probability.

## RESULTS

The demographic data concerning the study subjects and the relationship of the positivity of AFB_1_-albumin adducts with these demographic characteristics are described in Table 1

**Table 1:**
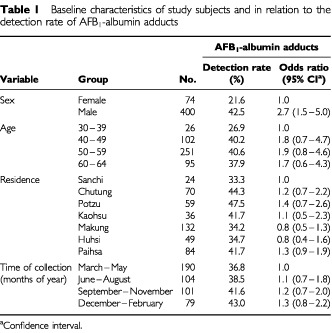
Baseline characteristics of study subjects and in relation to the detection rate of AFB_1_-albumin adducts

. There were no significant variation in detection rate of AFB_1_-albumin adducts among study townships (ranging from 33.3 to 47.5%), seasonality of sample collection (ranging from 36.8 to 43.0%) and age groups (ranging from 26.9 to 40.6%). In contrast, males had significantly higher detection rate of AFB_1_-albumin adducts than females (42.5 *vs* 21.6%) with an odds ratio (OR) of 2.7 (95% CI=1.5–5.0).

Results with regard to the detection rate of AFB_1_-albumin adducts in relation to multiple HCC risk factors are summarised in Table 2

**Table 2:**
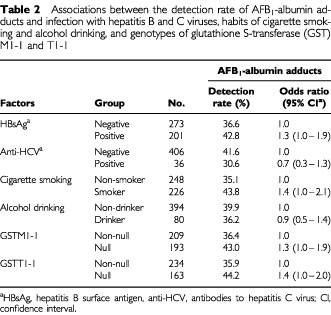
Associations between the detection rate of AFB_1_-albumin adducts and infection with hepatitis B and C viruses, habits of cigarette smoking and alcohol drinking, and genotypes of glutathione S-transferase (GST) M1-1 and T1-1

. AFB_1_-albumin adducts were detectable in 42.8% (86 of 201) of HBsAg carriers and 36.6% (100 of 273) of HBsAg non-carriers. The difference in the detection rate was moderately significant between the two groups (OR=1.3, 95% CI=1.0–1.9). This detection rate was higher in study subjects who smoked cigarettes (43.8%) than in those who never smoked cigarettes (35.1%), with a moderately significant OR of 1.4 (95% CI=1.0–2.1). In addition, there were also moderately significant differences in the adduct detection rate depending on *GSTM1-1* and *GSTT1-1* genotypes; the detection rate was higher in individuals with either *GSTM1-1* null (43.0%) or *GSTT1-1* null (44.2%) genotype than in those with *GSTM1-1* (36.4%) or *GSTT1-1* (35.9%) present. The OR of detectable AFB_1_-albumin adducts associated with *GSTM1-1* or *GSTT1-1* null genotype was 1.3 (95% CI=1.0–1.9) and 1.4 (95% CI=1.0–2.0), respectively. On the other hand, the detection rate was lower in anti-HCV-positive subjects (30.6%) than negative subjects (41.6%) (OR=0.7, 95% CI=0.3–1.3). There was again a slightly lower detection rate in individuals who consumed alcohol (36.2%) than in those who never drank alcohol (39.9%).

The effect of a combination of *GSTM1-1* and *GSTT1-1* genotypes on the detectable adduct levels was then analysed and stratified by HBsAg status. In this case the positivity of AFB_1_-albumin adducts tended to increase with increasing number of the null genotype of *GSTM1-1* and *GSTT1-1* in both the HBsAg carrier and non-carrier groups, albeit the trend was statistically non-significant (Table 3

**Table 3:**
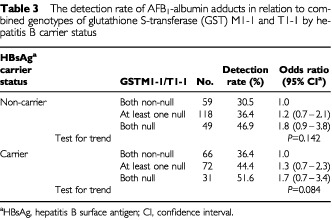
The detection rate of AFB_1_-albumin adducts in relation to combined genotypes of glutathione S-transferase (GST) M1-1 and T1-1 by hepatitis B carrier status

).

Results of logistic regression analysis of multiple factors associated with the positivity of AFB_1_-albumin adducts are shown in Table 4

**Table 4:**
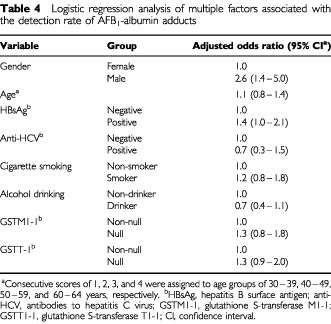
Logistic regression analysis of multiple factors associated with the detection rate of AFB_1_-albumin adducts

. After multivariate adjustment, male gender and positive HBsAg status was still significantly associated with positivity of AFB_1_-albumin adducts (for males: adjusted OR=2.6, 95% CI=1.4–5.0; for HBsAg carriers: adjusted OR=1.4, 95% CI=1.0–2.1). Whereas, no significant association with detectable AFB_1_-albumin adduct levels was observed for age, anti-HCV positive status, habits of cigarette smoking and alcohol intake, and the null genotype of *GSTM1-1* and *GSTT1-1*.

## DISCUSSION

Data from human epidemiological studies have demonstrated that exposure to AFB_1_ is one of the major risk factors in the multifactorial etiology of HCC (Ross *et al*, 1992; Mcglynn *et al*, 1995; Chen *et al*, 1996a,b; Wang *et al*, 1996b; Sun *et al*, 2001). Many studies have further confirmed that the toxic and carcinogenic effects of the aflatoxins are manifested only after metabolism by members of the endogenous cytochrome P-450 enzyme superfamily (Guengerich *et al*, 1992; Gallagher *et al*, 1994). Based upon knowledge of AFB_1_ metabolism, a number of molecular dosimetry markers of aflatoxin exposure have been developed (Gan *et al*, 1988; Groopman *et al*, 1992; Yu *et al*, 1997). Among them the AFB_1_-albumin adduct has been a useful biomarker reflecting long-term exposure to aflatoxins in different populations (Gan *et al*, 1988; Wild *et al*, 1990) and linking to an elevated risk of HCC (Chen *et al*, 1996a,b; Wang *et al*, 1996b; Sun *et al*, 2001). Thus, exploring the determinants of formation of AFB_1_-albumin adducts may contribute to understanding the complex interaction among multiple risk factors involved in hepatocarcinogenesis.

The major factors determining the formation of AFB_1_-albumin adducts for an individual in this population are gender and HBsAg carrier status. Males have significantly higher detectable adduct levels than females. This finding is in contrast to those observed in previous studies whether there was no significant association between gender and aflatoxin adduct levels (Wang *et al*, 1996a; Wild *et al*, 2000). The gender difference in detectable adduct levels might be attributable to differences in aflatoxin exposure or metabolism. It has been noted that HCC is 2–3 times more frequent in males than in females in Taiwan, despite their similarity in HBsAg carrier status (Chen *et al*, 1997). The gender difference in the formation of AFB_1_-albumin adducts might contribute to the increased susceptibility to hepatocarcinogenesis in males.

In this study, there was moderately significant effect of HBsAg carrier status on detectable AFB_1_-albumin adduct levels. HBsAg-positive adults have higher detectable adduct levels than HBsAg-negative adults (42.8 *vs* 36.6%). In particular, HBsAg-positive males have 50% higher frequency of detectable adducts than their HBsAg-negative counterpart (46.7 *vs* 39.5%; adjusted OR=1.5, 95% CI=1.1–2.4; data not shown). The possible association between chronic HBV infection and the increased activation of AFB_1_ has been examined in epidemiological studies. Two studies of young Gambian children have reported higher AFB_1_-albumin adduct levels in HBsAg carriers than in non-carriers (Allen *et al*, 1992; Wild *et al*, 1993). Another study of adolescents in Taiwan has also found a higher AFB_1_-albumin adduct levels in HBsAg-positive than negative subjects (Chen *et al*, 2001). Whereas, results from studies in adults are somewhat contradictory; in Guinea there was a non-significant increase in AFB_1_-albumin adducts in HBsAg-positive individuals (Diallo *et al*, 1995), while in China (Wang *et al*, 1996a) and Gambia (Wild *et al*, 2000) no such effect was observed. Furthermore, two recent studies of Chinese adult populations revealed that HBsAg positive status did not have significant effects on the temporal variability in AFB_1_-albumin adducts (Wang *et al*, 1996a; Ahsan *et al*, 2001). Therefore, we are left with apparent inconclusive results between studies in children and adults as well as between studies in adults in different populations regarding the effect of chronic HBV infection on AFB_1_-albumin adduct levels. In this study, a moderately significant difference in detectable AFB_1_-albumin adduct levels was found between HBsAg-positive and negative adults, particularly in males. This finding may result in part from the selection of study subjects from individuals who participated in a liver cancer screening programme. Those subjects may have viral-associated underlying subclinical liver diseases and may have a significant stimulation of the activation of AFB_1_, as indicated in previous studies (de Flora *et al*, 1985). Although it is known from experimental studies that liver injury associated with HBV can affect expression of carcinogen metabolizing enzymes (de Flora *et al*, 1989; Chemin *et al*, 1999), the biological mechanism that underlies the interaction between chronic HBV infection and increased activation of AFB_1_ in humans merit further studies.

Accumulating evidence indicates that genetic polymorphisms in AFB_1_ metabolising enzymes are a factor in individual susceptibility to aflatoxin-induced hepatocarcinogenesis (Mcglynn *et al*, 1995; Chen *et al*, 1996b; Sun *et al*, 2001). Members of the glutathione S-transferase (GST) family, such as GST-μ (*GSTM1-1*) and GST-θ (*GSTT1-1*), are important candidates for involvement in susceptibility to aflatoxin-related liver cancer because they may regulate an individual's ability to metabolize the ultimate carcinogen of aflatoxins, the exo-epoxide (Johnson *et al*, 1997). The current study observed that the detection rate of AFB_1_-albumin adducts tended to increase as the number of null genotypes of *GSTM1-1* and *GSTT1-1* increased. This biological gradient was observed in both HBsAg carriers and non-carriers, albeit the trend was statistically non-significant.

In essence, our knowledge base about determinants of formation of AFB_1_ macromolecular adducts in humans is still limited. In this study, we have assessed the relative contributions of environmental determinants and host susceptibility factors in the formation of aflatoxin–albumin adducts in a well characterised Chinese adult population. The result of present study suggests that gender and HBsAg carrier status are major determinants of the formation of aflatoxin covalently bound to albumin. This study further emphasises the necessity to reduce aflatoxin exposure in people living in an area endemic for chronic HBV infection.
